# Addition of Dexmedetomidine and Fentanyl to Intrathecal Hyperbaric Bupivacaine for Lower Limb Surgeries: A Randomized, Comparative Study

**DOI:** 10.7759/cureus.28276

**Published:** 2022-08-22

**Authors:** Jitendra V Kalbande, Ketki D Deotale, Archana K N, Habib Md R Karim

**Affiliations:** 1 Anaesthesiology and Critical Care, All India Institute of Medical Sciences, Raipur, Raipur, IND; 2 Anaesthesiology, JSS (Jagadguru Sri Shivarathreeshwara) Medical College and Hospital, Mysuru, IND

**Keywords:** quality, acute pain, dexmedetomidine, fentanyl, bupivacaine, spinal anesthesia

## Abstract

Background and aim: There is an unmet need to prolong analgesia duration following regional anesthesia; dexmedetomidine as an adjuvant for the intrathecal block has gained popularity over the last few years. The present study compares the onset, duration of sensory and motor block, postoperative analgesia, hemodynamic changes, and adverse effect of dexmedetomidine or fentanyl as an adjuvant to hyperbaric bupivacaine administered intrathecally.

Methods: With approvals, 60 American Society of Anesthesiologists (ASA) physical status I and II adult patients undergoing lower limb surgeries under subarachnoid block were randomized to receive either 5 µg dexmedetomidine (group BD, n=30) or 25 μg fentanyl (group BF, n=30) intrathecally along with 12.5 mg hyperbaric bupivacaine. The time to onset of sensory and motor blockade, time to peak block, intraoperative hemodynamic variations, duration of postoperative analgesia, and associated intraoperative and postoperative complications, if any, were recorded and compared statistically. SPSS v16 (IBM Corp., New York, United States) was used, and P<0.05 was considered significant.

Results: The onset of sensory block in group BD was 1.54 ±0.38 minutes and 3.4 ± 0.40 minutes (P<0.001) in group BF. Time taken for the sensory level to reach T10 in group BD was 3.11± 0.43 minutes and 5.55 ± 0.60 minutes (P<0.001) in group BF. Time taken for two-segment regression in group BD was 160.06 ± 6.85 minutes and 110.4 ± 6.03 minutes (P<0.001) in group BF. The onset of motor block was 2.58 ± 0.437 minutes in group BD and 4.43 ± 0.43 minutes (P<0.001) in group BF. The total duration of analgesia in group BD was 365.8 ± 24.76 minutes and 213.33 ± 20.19 minutes (P<0.001) in group BF. Minimum intraoperative hemodynamic variations were found in group BD, and two groups had comparable side effects.

Conclusion: Dexmedetomidine 5 μg added to intrathecal bupivacaine produced early-onset and prolonged block compared with fentanyl 25 μg. No significant attributable adverse effects were noted for both the drugs except the fall in blood pressure, which was gradual in dexmedetomidine but a steep fall in fentanyl.

## Introduction

The increased incidence of trauma in the present day has resulted in increased orthopedic surgeries, often associated with severe postoperative pain. Decreased mobility postoperatively caused by severe pain impairs early ambulation and increases the risk of thromboembolism. Postoperative pain relief is a growing concern for anesthesiologists as an uneventful postoperative period makes surgery a comfortable proposition for surgical patients [1].

A subarachnoid block is a relatively safe anesthetic method; it also has the advantage of providing postoperative pain relief, with preservation of mental status and normal airway reflexes. The use of adjuvants may reduce the nature of complications and improve the duration of the anesthetic effect. Some commonly used adjuvants are opioids, ketamine, neostigmine, clonidine, and dexmedetomidine.

The aim of using neuraxial opioids is to achieve analgesia comparable to systemic administration, but with significantly smaller doses. Fentanyl is primarily a µ-receptor agonist. When administered intrathecally, the time to onset of its action is five minutes and the duration of action is three to five hours. The significant advantage of "selective spinal analgesia" by fentanyl lies in the absence of sympathetic blockade and postural hypotension, potentially allowing early ambulation of the patient and avoidance of cardiovascular collapse or convulsions, which are major complications of a spinal anesthetic blockade [2].

Dexmedetomidine is an α2-adrenergic receptor (α2-AR) agonist which prolongs the duration of both sensory and motor blockade induced by local anesthetics irrespective of the route of administration (e.g., epidural [3], caudal [4], or spinal [5]). When given intrathecally, it acts at the spinal and supraspinal levels. It activates α2-AR in the spinal cord, reducing transmission of nociceptive signals, inhibiting substance P's release, and contributing to their analgesic action, and has a significant opioid-sparing effect [6]. At the supraspinal level, it binds to the presynaptic α2-ARs in locus ceruleus, producing sedation and anxiolysis; postsynaptic activation in CNS inhibits sympathetic activity leading to a decrease in heart and blood pressure. Rapid administration of dexmedetomidine infusion (loading dose of 1 μ kg^-1^ if given in less than 10 minutes) may cause transient hypertension mediated by peripheral α2B-adrenoreceptor vasoconstriction [7]. The present study aims to evaluate the efficacy of intrathecal administration of dexmedetomidine in providing intraoperative and postoperative analgesia and hemodynamic stability, as compared to intrathecal fentanyl, when used as adjuvants to hyperbaric bupivacaine.

## Materials and methods

Study settings and population

In this randomized clinical trial, 60 patients from the age group of 20-70 years belonging to the American Society of Anesthesiologists (ASA) physical status І and ІІ and posted for elective lower limb orthopedic surgeries were studied at an academic institute in Mysore, India, between November 2010 and May 2012. Institute ethical committee permission was taken before starting the study.

Ethics approvals, patient consent, and trial registration

The study was evaluated and approved by the Post-graduate Thesis Review Committee and Institutional Ethics Committee of JSS Medical College and Hospital, Mysore, India. Clinical Trial registration was not done.

Randomization, allocation, and blinding

Patients were randomized using a random number table provided by a statistician. The study population was divided into two groups of 30 each: group BF, which received 2.5 mL of bupivacaine (hyperbaric) + 25 µg fentanyl in 0.5 mL, and group BD, which received 2.5 mL of bupivacaine (hyperbaric) + 5 µg dexmedetomidine in 0.5 mL. The group allocation was performed by the principal investigator. Allocation concealment was done by keeping the random numbers sealed and was only opened by the principal investigator. The drugs were prepared by the principal investigator, and the names of the drugs were not marked on the syringe to maintain double-blinding.

Patient selection and intervention

Patients with a history of known sensitivity to the drugs used and those with gross spinal deformity, peripheral neuropathy, or contraindications to neuraxial block were excluded. A thorough pre-anesthetic evaluation with the general, systemic, and spine examination was done the evening before surgery. A routine investigation was carried out on all patients. After obtaining informed written consent for the study and the procedure, all the patients were prescribed 0.5 mg of alprazolam and ranitidine 150 mg orally the previous night. Patients were advised to be nil orally from midnight onward on the previous day of surgery. On the day of surgery, intravenous access was secured with an 18-gauge venous cannula; crystalloid was started. Non-invasive blood pressure, electrocardiogram, and pulse oximeter monitors were connected, and the baseline pulse rate, blood pressure, ECG, respiratory rate, and peripheral oxyhemoglobin saturation (SpO_2_) were recorded. All the blocks were performed by the same person. The patients and physicians evaluating the outcome of the treatments were blinded to the group allocation.

All patients were administered 500 mL of Ringer's lactate or normal saline before performing spinal anesthesia. The lumbar subarachnoid block was performed under strict aseptic precautions with the patients in the left lateral or the sitting position irrespective of the side of surgery. The spinal anesthesia puncture was done at the L3-L4 interspace, midline approach, using a 23- or 25-gauge (G) Quincke's needle after local infiltration of the skin using 2% xylocaine. After confirming a free and clear flow of CSF, the study drug was administered slowly. Patients were made to lie supine immediately. The time of injection of the drug was recorded as the zero timepoint. Intraoperative intravenous fluids (normal saline and Ringer lactate) were at the discretion of the anesthesiologists. Intraoperatively, non-invasive blood pressure (NIBP), ECG, and SpO_2_ were recorded every two minutes for the first 10 minutes, followed by every five minutes for the rest of the surgery. The time interval at which hypotension, bradycardia, or other complications occurred was noted. Oxygen 5 L/min via face mask was administered to all patients throughout the procedure.

Outcome measures

The study was planned with the primary objective of determining the duration of postoperative analgesia provided by the adjuvants. The other secondary objectives were to find and compare the onset of sensory, motor, intraoperative hemodynamics variation, and block regression times. The following parameters have been noted: the onset of sensory block, the time required to reach T10 level, maximum level achieved, and duration of analgesia. The onset of sensory block was taken as the time from deposition of a drug to the feeling of tingling sensation in the legs. Sensory anesthesia was defined as loss of sharp sensation to pinprick bilaterally in the midclavicular line started at T10, which was tested with a 23-G blunt tip hypodermic needle. Peak sensory level was defined as the sensory level which remained the same for three readings taken after a one-minute interval. The onset of motor block was noted as the time taken from onset of paresis to the loss of power, i.e., motor blockade at the level of hip and knee. The patients were monitored for untoward effects such as inadequate block, hypotension, bradycardia, respiratory distress, nausea, vomiting, restlessness, pruritus, shivering, and anaphylactic reaction intraoperatively. Pain relief was assessed using visual analog scores (VAS), for the determination of the need for rescue analgesia only which was provided with diclofenac sodium 1.5 mg/kg body weight when the VAS score was found to be more than or equal to 4 or on demand. The duration of analgesia was defined as the duration from the end of the surgery to the time of the first need for rescue analgesia. Patients were shifted to the postoperative ward and observed till the administration of analgesic (diclofenac sodium 1.5 mg/kg, intramuscularly as per the patient's demand) and for the next 72 hours post-operatively for delayed complications.

Hypotension was defined as a decrease in systolic blood pressure by 25% of baseline value or any value below 90 mmHg. Hypotension was treated with injection (Inj.) mephentermine intravenously at 6 mg increments. Bradycardia was defined as heart rate <60/minute and was treated with Inj. atropine 0.6 mg IV when associated with hypotension also. Simultaneously nausea and vomiting were treated with Inj. ondansetron 4 mg IV. Shivering was treated with warm drapes and warm intravenous fluids.

Sample size calculation

The sample size for the study was calculated for a hypothesized difference in the postoperative analgesia duration by 25% between the groups. We considered 80% power, and the sample was calculated for 95% confidence which gave a sample of 26 patients per group. However, we decided to recruit 30 participants per group.

Statistical analysis

The discreet integral data are presented in absolute number and percentage scales. Continuous data are presented in mean and standard deviation; the independent-samples t-test procedure compares means for two groups of cases. General linear model repeated measures were used for analyzing the effect over different times. The categorical data between study groups were compared by using Fisher's exact test as appropriate. All the statistical calculations were done through SPSS 16.0 (2007) (IBM Corp., New York, United States) for windows; P-values of less than 0.05 were considered statistically significant.

## Results

No patients required exclusion after recruitment and data from the entire 60 patients who were eligible for inclusion were analyzed. The two groups were statistically similar to each other in age, gender, ASA physical status, type of surgery, and duration of surgery (Table 1). The mean duration of surgery in group BD was 101.16 minutes and 97.66 minutes in group BF. Common procedures done were open reduction with internal fixation for fracture femur, both lower limb bones, debridement, and implant removals; the distribution of the procedures among the groups was even (contingency coefficient: 0.203, P<0.99). There were statistically significant differences between groups BD and BF in the time of onset of sensory block (P<0.001), time taken to reach T10 level (P<0.001), the onset of motor block (P<0.001), time taken for two-segment regression (P<0.001), and duration of postoperative analgesia (P<0.001). The difference in characteristics of the block between the two groups is presented in Table 1.

**Table 1:** Comparison of demographic, surgical duration, and block characteristics between the groups ASA PS: American Society of Anesthesiologists Physical Status, BD: bupivacaine + dexmedetomidine, BF: bupivacaine + fentanyl.

Parameters	BD Group (n=30)	BF group (n=30)	P-Value
Mean age (years)	43.8	46.4	0.863
Male	24 (80%)	21 (70%)	0.371
Female	6 (20%)	9 (30%)	0.371
ASA PS-I	13 (43.3%)	12 (40%)	0.793
ASA PS-II	17 (56.6%)	18 (60%)	0.793
Mean duration of surgery (min)	101.16	97.66	0.594
Time from injection to the onset of sensory block (min)	1.54 ± 0.38	3.4 ± 0.40	P<0.001
Time required for the sensory level to reach T10 (min)	3.11 ± 0.43	5.55 ± 0.60	P<0.001
The onset of motor block (min)	2.58 ± 0.43	4.43 ± 0.43	P<0.001
Time required for two-segment regression of sensory block (min)	160.06 ± 6.9	110.4 ± 6.03	P<0.001
Duration of postoperative analgesia (min)	365.8 ± 24.7	213.3 ± 20.2	P< 0.001

The VAS score was only used to determine the time until the requirement of rescue analgesia (i.e., a VAS score of 4 or more). In group BF, most patients (96.7%) required rescue analgesia before 300 minutes, while a majority of patients in group BD (90%) required rescue analgesia between 300 and 400 minutes. The mean duration of analgesia in group BF was 213.33 minutes and in group BD was 365.8 minutes. In group BD, the majority of patients (46.7%) achieved the maximum sensory block level of T10, whereas, in group BF, the maximum number of patients (53.3%) reached a maximum height of T8. No statistically significant difference was observed between the two groups (P<0.715). However, there were a few patients with levels progressing further to the highest sensory level of T6 in both groups (Table 2).

**Table 2:** The distribution of the height of sensory blockade achieved for the dermatome T: thoracic, T6: thoracic 6 dermatome, BD: bupivacaine + dexmedetomidine, BF: bupivacaine + fentanyl.

Max Level Reached	Group BD (n=30)	Group BF (n=30)
T6	3 (10%)	3 (10%)
T8	13 (43.3%)	16 (53.3%)
T10	14 (46.7%)	11 (36.7%)
Total	30 (100%)	30 (100%)

**Figure 1:**
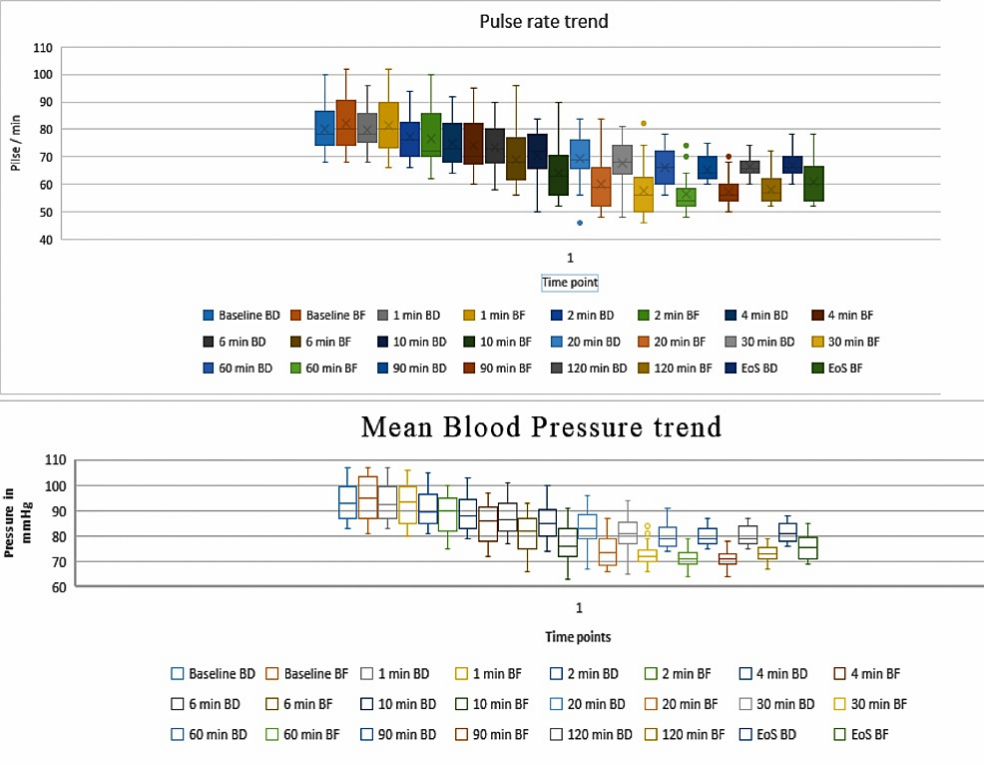
Box and Whisker plot showing the trend of pulse rate and mean blood pressure among the groups The timepoints are in minutes. Box and Whisker show maximum, minimum, mean, and interquartile ranges. EoS: end of surgery, BD: group bupivacaine plus dexmedetomidine, BF: group bupivacaine plus fentanyl.

Both pulse rate and blood pressure changes had a steeper fall in group BF, whereas, in group BD, there was a gradual fall as depicted in Figure 1.

Changes or falls in mean pulse rate (PR), systolic blood pressure (SBP), and diastolic blood pressure (DBP) were gradual in group BD, and after some time (i.e., 10 to 15 minutes after spinal anesthesia), it achieved a steady state, which was maintained till the end of surgery. Changes and falls in mean PR, SBP, and DBP were higher in group BF and were statistically significant (P<0.001). About 6%-10% of the patients had nausea, vomiting, and pruritus in the group BF as compared to none in the group BD; however, intraoperative complications between the two groups were found to be statistically indifferent (Table 3).

**Table 3:** Intraoperative complications between the groups BD: bupivacaine + dexmedetomidine, BF: bupivacaine + fentanyl.

Complications	Group BF (n=30)	Group BD (n=30)	P-Value
No complication	16 (53.33%)	26 (86.66%)	P<0.837
Hypotension	2 (6%)	1 (3%)	
Bradycardia	2 (6%)	1 (3%)	
Hypotension + bradycardia	2 (6%)	1 (3%)	
Shivering	2 (6%)	0 (0%)	
Nausea + vomiting	3 (10%)	1 (3%)	
Pruritus	3 (10%)	0 (0%)	
Total	30 (100%)	30 (100%)	

## Discussion

The present study finding suggests that both fentanyl and dexmedetomidine can be safely and effectively used as an adjuvant to intrathecal bupivacaine for spinal anesthesia. Both drugs achieved a similar level of blockade and produced almost similar side effect profiles. However, dexmedetomidine at a dose of 5 µg showed a better profile for the duration of blockade and time to the requirement for postoperative rescue analgesics. Further, the hemodynamic profile and fall pattern were also favorable as compared to fentanyl 25 µg as an adjuvant. Additionally, patients receiving dexmedetomidine had a faster onset of sensory action (1.54 ± 0.38 minutes) which was statistically significant. Our findings are consistent with those of Khosravi et al. [8] and of Shukla et al. [9] studies that also found a better onset time for 5 µg dexmedetomidine.

In the present study, dexmedetomidine 5 µg as an adjuvant has provided a prolonged duration of analgesia in the form of sensory blockade, up to 365 minutes, reducing the need for rescue analgesics and polypharmacy in the postoperative period. Paramasivan et al. [10] in a systemic review and meta-analysis on the effect of intrathecal dexmedetomidine found a similar finding, i.e., median analgesic duration in the dexmedetomidine group was 363.6 minutes (range: 252.3-824.0) compared to the placebo group. Rahimzadeh et al. and others also mentioned a mean duration of analgesia longer than 250 minutes with 5 μg dexmedetomidine compared to 25 μg fentanyl [11-12]. A mechanism for prolonged analgesia is that intrathecal α2-adrenoceptor agonists potentiate the effects of intrathecal local anesthetics by depressing the release of neurotransmitters by presynaptic C-fibers and by hyperpolarization of postsynaptic dorsal horn neurons [5].

The other interesting finding in our study was that the intrathecal dexmedetomidine group exhibited better hemodynamic stability than the fentanyl group, which was statistically significant. Although there was a fall in blood pressure in both groups, the fall was not abrupt in dexmedetomidine. This finding is, however, in contrast to the other findings of the studies conducted by Kanazi et al. [5] and Gupta et al. [11], and others [10,13] did not observe any significant difference between the groups for hemodynamic stability.

Although a good number of studies have addressed the administration of different doses of intrathecal dexmedetomidine (3 μg, 5 μg, 10 μg, and 15 μg) as an adjuvant to local anesthetics [14-16], the ideal dexmedetomidine dose is yet to find. To achieve better efficacy, we can increase the dose of the used dexmedetomidine. Gupta et al. [17] reported that increasing the dose of dexmedetomidine from 2.5 μg to 10 μg would show better and longer sensory and motor block, with a longer duration of anesthesia and comparable hemodynamic and side effects profile. However, the possibility of deteriorated hemodynamics with increasing doses cannot be ruled out yet.

Recently, systematic reviews and meta-analyses have evaluated the effect of the addition of dexmedetomidine 5 μg to bupivacaine for spinal anesthesia and have found that it prolongs both sensory and motor blockade duration and increases the time for rescue analgesic requirement after surgery [18]. Another systematic review and meta-analysis has compared the addition of dexmedetomidine and fentanyl as an adjuvant to local aesthetics for abdominal hysterectomies [19]. They found that both agents were similar in efficacy in terms of attaining the sensory blockade; however, the dexmedetomidine group had better visceral pain control and was better efficacious than fentanyl in terms of the requirement of medication for visceral pains [19].

Choosing a medication needs consideration for the highest efficacy yet having the lowest side effects. In our study, although the overall side effect profiles were similar among the groups, the dexmedetomidine group had no pruritus or postoperative nausea vomiting (PONV) as compared to fentanyl. Although insignificant, it bears clinical importance as both pruritus and PONV are bothering the patient and affect satisfaction.

The study’s main limitation was that it involved relatively healthy adults, and the effect on older patients with cardiovascular morbidities is unknown. Secondly, total analgesic consumption in 24 hours was not noted. Our study is well-powered for postoperative analgesia duration, but not for the other findings like the better profile of PONV and pruritus noted in the dexmedetomidine group. Further well-powered studies for adverse effects will be required to determine the superiority of dexmedetomidine in these aspects. The other limitation of the study is the limited use of VAS scores across timepoints. As we decided only to find the duration of analgesia provided, we used the VAS score to find out the requirement of rescue analgesia only. We have not collected the VAS score beyond that point and therefore could not compare them among the groups.

## Conclusions

Dexmedetomidine 5 μg added to intrathecal bupivacaine produced early-onset and prolonged block compared with intrathecal fentanyl 25 μg. No significant attributable adverse effects were noted for both the drugs except the fall in blood pressure, which was gradual in dexmedetomidine but a steep fall in fentanyl. A better profile of PONV, shivering, and pruritus was noted in the dexmedetomidine group. However, our study is underpowered for adverse effects, and further studies will be required to determine the superiority of dexmedetomidine in these aspects.
